# Bullous pemphigoid in diabetic patients treated by gliptins: the other side of the coin

**DOI:** 10.1186/s12967-021-03192-8

**Published:** 2021-12-20

**Authors:** Karim Chouchane, Giovanni Di Zenzo, Dario Pitocco, Laura Calabrese, Clara De Simone

**Affiliations:** 1 Whittington Health NHS Trust, Magdala Ave, London, N19 5NF UK; 2 Molecular and Cell Biology Laboratory, Istituto Dermopatico dell’Immacolata (IDI) IRCCS, Rome, Italy; 3 Diabetes Care Unit, Endocrinology, University Hospital “A. Gemelli”, Catholic University of the Sacred Heart, Rome, Italy; 4 Institute of Dermatology, University Hospital “A. Gemelli”, Catholic University of the Sacred Heart, Rome, Italy; 5 Department of Dermatology, Fondazione Policlinico Universitario A. Gemelli IRCCS, Rome, Italy

**Keywords:** Type-2 diabetes, Gliptins, Dipeptidyl peptidase-4 inhibitors, Bullous pemphigoid

## Abstract

Bullous pemphigoid (BP) is the most common autoimmune bullous skin disease that affects primarily patients older than 60 years. The majority of BP cases are spontaneous, but BP can also be triggered by certain drugs’ exposures. Since 2011, a growing number of observations has been reporting cases of BP in Type 2 diabetic patients. These forms have been linked to the use of a new category of anti-diabetic drugs called dipeptidyl peptidase inhibitors (DPP-4i) or gliptins, but to date, the exact pathophysiological mechanisms underlying this association are not completely elucidated. Although conventional and gliptin-associated BP are thought to share similar clinical and histopathological features, our thorough review of the most recent literature, shows that these 2 forms are quite distinct: DPP-4-i-associated BP seems to appear at an earlier age than spontaneous BP, it may manifest either as a noninflammatory or inflammatory phenotype, while the conventional form presents with a typical inflammatory phenotype. Additionally, an important distinctive histological feature was recently shown in Gliptin-associated BP: these forms may present a less significant eosinophils infiltrate in the upper dermis of peri-blister lesions compared to the skin of patients with spontaneous BP, and this seems a specific feature of the clinically non-inflammatory forms. In accordance with previous literature, we found that the direct immunofluorescence (DIF) gives identical findings in both DPP-4i-associated and conventional forms of BP which is an IgG and complement C3 deposition as a linear band at the dermal–epidermal junction in perilesional skin. Indirect immunofluorescence shows the presence of IgG circulating autoantibodies in the patient's serum which titer does not differ between spontaneous and DPP-4i-associated BP, while the specificity of these autoantibodies, may be different in spontaneous, induced non-inflammatory and induced inflammatory forms, epitope spreading phenomenon seems to play a role in determining these specificities. Further research, based on integrated epidemiological, clinical, histo-immunological and pharmacogenomic approaches, may give more insight into these forms of BP. This combined approach will allow to better define BP endotypes and to unveil the mechanism of spontaneous or drug-induced breakage of the immunotolerance to skin self-antigens.

## Background

Bullous pemphigoid (BP), first described as an independent entity by Lever in 1953 [1], is the most common autoimmune bullous skin disease. It is an acquired, chronic, subepidermal bullous disease caused by the production of autoantibodies against hemidesmosomal components of basal keratinocytes. BP affects primarily patients older than 60 years, and classically manifests by intense pruritus, tense blisters, and erosions over urticarial plaques on the trunk, extremities and face, with an uncommon oral mucosal involvement [2–5].

The pathogenesis of BP can be explained by the presence of a dysregulated T cell immune response and the synthesis of IgG and IgE autoantibodies against hemidesmosomal proteins (BP180 and BP230) involved in the dermal-epidermal cohesion, leading to neutrophil chemotaxis and degradation of the basement membrane zone.

The diagnosis of BP relies on clinical assessment and positive direct immunofluorescence microscopy. Other assays have confirmatory value such as histopathological evaluation, indirect immunofluorescence assays and the quantification of circulating autoantibodies against BP180 and/or BP230 using ELISA [6].

BP affects predominantly elderly persons, with a mean age of onset ranging from 73 to 88 however, exceptional pediatric cases were reported [7, 8]. BP has a higher incidence in females and is associated with significant morbidity and mortality.

Various comorbidities have been reported with BP such as neurological disorders including Parkinson’ disease, multiple sclerosis, and dementia, thrombotic disorders, and hematologic malignancies [3, 8, 9]. BP has been reported in association with certain skin diseases such as psoriasis and lichen planus that seems to trigger BP through an immune response due to the chronic inflammation at the dermal–epidermal junction, eventually leading to autoimmunity [3]. BP can also be triggered by certain drugs exposures [10]. Currently, gliptins, also known as dipeptidyl peptidase-4 inhibitors (DPP-4i), a new family of glucose lowering agents, are at the forefront of medications inducing BP. A growing amount of literature has been recently addressing the association BP-Gliptins and the potential mechanisms that may drive this association, but to date, the exact pathophysiological mechanisms underlying this association are not completely elucidated.

In the present review, we will revisit the drug-induced BP with focus on the gliptins-related forms. We will study the mode of action of gliptins, and discuss the suggested mechanisms explaining the relationship between these drugs’ usage and the occurrence of BP. We will also study the distinctive clinical, biological and immunological features of gliptins-induced forms of BP.

## Bullous pemphigoid’s incidence is increasing worldwide

The yearly incidence of BP ranges in European countries from 2.5 to 42.8/million, with an obvious increasing trend noted during the recent years in all studies [3, 11]. In France, the BP incidence previously estimated at 7 new cases/million/year in 1995 [12], increased to 21.7 cases/million/year during the period 2000–2005 [8]. This increasing trend reported in Europe was echoed in other parts of the World: the incidence of 7.6 cases/million/year reported in Israel in the period 2000–2005, increased to 14.4 cases between 2011–2015 [13], and it is estimated that globally, an overall increase of 1.9 to 4.3-fold in BP incidence rates occurred during the past 2 decades [14, 15]. This higher incidence seems to be attributed to aging populations, improvement in the diagnosis of the non-bullous presentations of the disease, and to drug-induced cases [16].

During the last decades, an increasing number of observations has been reporting cases of BP in diabetic patients [17], and more insight was obtained by epidemiological studies, linking those BP cases in diabetic patients to the use of a new category of drugs which are the dipeptidyl peptidase inhibitors (DPP-4i) or gliptins, extensively used in the treatment of type 2 diabetes mellitus (T2DM). Since then, a considerable amount of literature is shedding light into the association between DPP-4i use and BP.

## Drug induced BP (DIBP), causatives drugs

Drug induced BP (DIBP), is an entity characterized by similar clinical, pathological, and immunological features than the conventional BP, which appears in association with the systemic intake of particular drugs [18]. Unlike spontaneous BP, DIBP occurs in younger patients, it may persist for up to several months after drug discontinuation, and is characterized by rare relapses [19]. At least 50 and up to 90 drugs have been to date implicated in the induction of BP [17, 18, 20]. Sufficient data supports presently the associations of certain antibiotics (ampicillin, ciprofloxacin), ACE inhibitors (enalapril), diuretics (spironolactone, furosemide), statins, D penicillamine, tumor necrosis factor inhibitors, psychotropic drugs and analgesics with the occurrence of BP [17, 20–23]. With the advent of new therapies, allowing to tackle some of the most challenging diseases like cancers, more cutaneous drug-reactions are being reported. Cases of BP and mucous membranes pemphigoid are indeed increasingly observed with the use of certain checkpoint inhibitors (anti programmed death-1 (PD-1) and anti-programmed death-ligand-1 (PD-L1) therapies), new regimens used in cancer [24–27]. However, of all drugs families, gliptins seem to induce the highest risk of DIBP [16].

## Dipeptidyl peptidase-4 inhibitors

DPP-4i also known as gliptins, include Sitagliptin, Vildagliptin, Saxagliptin, Linagliptin, Alogliptin and Anagliptin. These are glucose lowering agents whose tolerability, safety and good efficacy in diabetes type 2, as monotherapy or in combination with other oral antidiabetic agents or with insulin, are largely demonstrated [28–30]. First approved for use in diabetes in 2006 [31], DPP-4i are currently recommended as second or third-line therapies in all guidelines for the management of T2DM [28]. They may also be indicated as first-line medication in case of metformin contra-indication or intolerance, and in patients with advanced disease in combination with basal insulin therapy. As add-on therapy or monotherapy, gliptins are well tolerated and do not adversely affect Beta cell survival. Additionally, they promote glycemic control without increasing hypoglycemia risk, which is seen as a significant advantage, since hypoglycemia episodes were linked to major cardiovascular events in diabetic subjects [32]. Finally, gliptins are weight neutral and safe in elderly patients even in case of diabetic nephropathy [29].

DPP-4i are currently commonly prescribed for T2DM, Plaquevent et al. studied the observed rate of gliptin intake in a large sample of 1787 BP patients in comparison with the expected rate after indirect age standardization on 225,412 individuals from the database of the National Healthcare Insurance Agency. The rate of intake of gliptins and that of vildagliptin was higher than expected in BP patients, with an observed-to-expected drug intake frequency ratio of 1.7 for the whole gliptin class and 4.4 for vildagliptin [33, 34].

### Mechanism of action of DPP-4i

DPP-4i are competitive reversible inhibitors of the DPP-4 enzyme acting extra-cellularly [35]. Their specific target, the DPP-4 enzyme, also known as CD26 [16, 36], is a type II transmembrane glycoprotein that exists as a soluble form in body fluids [35], and which is ubiquitously expressed on the surface of a big variety of cells [29, 37]. DPP-4 belongs to the group of serine proteases, it selectively cleaves N-terminal dipeptides from a variety of substrates including neuropeptides, cytokines and incretins. This enzymatic cleavage may disactivate certain substances, regulate the actions of others, and may also induce the release of bioactive substances with novel effects [31, 37]. The effect of DPP-4i in diabetes is mediated by their action on incretins. Incretin hormones, namely, glucagon-like peptide-1 (GLP-1) and glucose-dependent insulinotropic polypeptide (GIP) are naturally occurring glucoregulatory hormones which are secreted, in a glucose-related manner, by the endocrine cells of the gut immediately after meal intake [29]. Incretins increase insulin release and suppress glucagon production by targeting specific receptors on Beta and alpha cells of the pancreas [38, 39] but are rapidly degraded by DPP-4. Gliptins inhibit the enzymatic activity of DPP-4 blocking consequently the degradation of GIP and GLP-1. The gliptin-induced elevation of incretins level, extends the post-prandial insulin secretion and action and inhibits glucagon secretion, enhancing, thus, the blood glucose homeostasis [31, 35, 38, 40].

DPP-4 is not specific for insulinotropic hormones, it is ubiquitously expressed in various cells including immune cells, fibroblasts, epithelial cells, stromal and stem cells, and in various organs such as pancreas, liver, spleen and gastrointestinal tract, which explains its pleiotropic role beyond incretin degradation [29, 41, 42]. DPP-4 is also expressed in subcutaneous and visceral adipose tissues and it has been recently shown that the level of circulating soluble form of DPP-4, identified as adipokine, is increased in obesity and type 2 diabetes. This adipokine could be a marker of vascular dysfunction and a potential molecular link between, obesity, diabetes and cardiovascular morbidity [31, 38]. Moreover, it has been indicated that other substrates of DPP-4, such as neuropeptide Y and substance P, are involved in cardiovascular regulation, feeding regulation, and mediation of pain [43]. From another hand, DPP-4 is enzymatically active in both soluble and membrane-bound forms which confers to it further regulatory effect on a large variety of substances involved in body homeostasis regulation [43]. Finally, in addition to its enzymatic action, DPP-4 can interact with various proteins, which extends its scope of actions to multiple biological processes such as cellular and humoral immunity, inflammation, and tissues remodeling [38, 44]: therefore, DPP-4 blockade is expected to induce a myriad of effects [41].

### Off-target effects of DPP-4i

It has been demonstrated that continuous blockade of DPP-4 might lead to an exacerbation of inflammatory processes [43]. On the other hand, DPP-4 is a member of a family of proteases including also DPP-8 and DPP-9 whose role is not yet completely defined [44]. Inhibition of these latter has been shown to induce severe toxicity in preclinical trials, therefore high selectivity of DPP-4i to DPP-4 is of utmost importance, to avoid such an off-target effect [45]. Some concerns were raised in post-marketing reports during the years following the commercialization of DPP-4i about the potential increase in the risk of acute pancreatitis and pancreatic cancer [28]. Subsequent trials showed that there was no evidence supporting the increase in pancreatic cancer risk, and several studies supported the safety of DPP-4i with regards to acute pancreatitis [46, 47]. Furthermore, there has been uncertainty about the long-term cardiovascular (CV) safety of DPP-4i. Outcome trial with saxagliptin showed an increased risk of hospitalization for heart failure in T2DM patients with established CV disease [48], although this effect was not confirmed in subsequent studies [29]. Conversely, several meta-analysis, randomized controlled trials and observational studies, compared DPP-4i to sulphonylureas (Sus), another glucose-lowering agent, with and without the addition of metformin. They reported lower rates of total adverse events, major adverse cardiovascular events, non-fatal CV events, CV mortality and all-cause mortality with DPP-4i usage [49–51]. Finally, a few observations reported the occurrence of arthralgia with gliptins use, though the causality is not yet established [28].

## DPP-4i-induced BP

### Evidence for the role of DPP-4i in BP induction

Pasmatzi et al. [52] and Skandalis et al. [53] first drew the attention in 2011–2012 to the association gliptins-BP by reporting cases of BP occurring in T2DM patients receiving a gliptin (mostly Vildagliptin) in conjunction with metformin. Those cases did not seem to be coincidental nor metformin-induced, since T2DM patients do not have a particular susceptibility for BP, and the metformin, in use for several decades, was not shown to induce such a reaction. Additionally, the temporal relationship between the introduction of the gliptin agent and the manifestation of BP, and its remission after withdrawal of the drug with mild therapeutic intervention, led to the conclusion that DPP-4i are the culprit agents. Since then, the association of DPP-4i and BP has been continuously reported through case reports, small case series [54–61], observational studies [33], and pharmacovigilance database analysis [34]. In a recent retrospective case–control study, DPP-4i are associated to threefold increase of BP risk. Multivariate logistic regression analysis confirmed DPP-4i as independent risk factor for BP after adjustment for potential confounding variables susceptible of increasing BP risk such as the co-existence of a neurological disease [14]. Varpuluoma et al. found, in a retrospective study including diabetic patients with BP on DPP-4i, and diabetic patients with BP not exposed to DPP-4i, that DPP-4i exposure is responsible for a 2.2-fold increase in the risk of BP [62]. A systematic review and meta-analysis including 3563 patients indicated a 3.6-fold increased risk of BP in patients receiving DPP-4i [63]. A recent large population-based study in UK, indicated that the use of DPP-4i was associated with at least a doubling of the risk of BP in patients with T2D compared to the general population [64]. All DPP-4i have been reported in association with BP, suggesting a class effect, but through the majority of studies, vildagliptin was showing the strongest association with BP followed by linagliptin which was found significantly implicated in BP occurrence [14, 34, 62]. A Finnish nationwide registry study reported a 10 folds increased risk of BP with vildagliptin use [62]. In line with these results, Phan et al. confirmed, in a recent systematic review and an adjusted meta-analysis including five retrospective case control studies, the higher risk with vildagliptin compared to linagliptin and did not show an association between sitagliptin and BP [65]. This association involving particularly vildagliptin has also been observed in two pharmacovigilance studies which found an alarming number of BP associated with vildagliptin compared to other drugs [34, 66]. But to date, no explanation was found to the higher responsibility of vildagliptin in the occurrence of BP, compared to other gliptins [34]. Of note, none of the other oral glucose-lowering agents was associated to an increased BP risk [14, 62, 67].

### Population at risk

The association DPP-4i and BP was stronger for male patients in certain studies [14] and higher for female patients in others [62]. An equally significant association DPP-4i-BP in both males and females was reported by the vast majority of studies [64]. The age of patients treated with DPP-4i does not seem to have a significant effect on the induction of a BP [62], although a higher risk was reported in patients aged below 70 years [14] and in those aged 80 years or above [68].

### Pathogenesis

The pathological mechanisms underlying the association between DPP-4i and BP have yet to be elucidated. As in conventional BP, the main features of DPP-4i induced BP are the disruption of the basal membrane and formation of blisters. These events are the result of the activation of several pathways including complement activation and deposition, neutrophilic chemotaxis, and release of proteases.

The triggering factor for this cascade is the binding of specific autoantibodies on their hemidesmosomal targets. In classical BP, autoantibodies (most commonly of the IgG isotype) and T cell response target two self-antigens namely BP180 (also called collagen XVII) and BP230 [3, 69]. These 2 proteins, components of hemidesmosomes, are present in basal keratinocytes and are responsible for the cohesion of the dermis and epidermis [3, 20, 70]. BP180 is a transmembrane glycoprotein whose juxtamembranous extracellular non collagenous domain 16A (NC16A) is the immunodominant epitope in BP. 80–90% of IgG autoantibodies in conventional BP target this extracellular NC16A domain, and their level is correlated with the severity of the disease [71, 72]. Epitope mapping studies have shown that NC16A domain itself harbors clusters of antigenic sites recognized by the majority of BP sera [73]. In conventional BP, IgG autoantibodies targeting epitopes other than NC16A domain, like C-terminal and intracellular epitopes, may be seen [16, 74, 75], mainly during early stages of the disease, or in association with mucosal involvement [76]. BP230 is an intracellular component of hemidesmosomes that belongs to the plakin proteins family; the N-terminal domain and the globular C-terminal domain of BP230 are the targets of IgG antibodies in BP [4]. In DPP-4i-induced BP, most of the IgG autoantibodies target preferentially epitopes in the mid portion of the extracellular domain of BP180 [74], including the LAD-1 and the C-terminal domain [54, 74], and not the juxtamembranous NC16A domain [16, 69, 74]. Nonetheless, IgG autoantibodies against BP 180 NC16A identical to those found in classical BP have been identified in certain cases of DPP-4i-induced BP [68]; it has been hypothesized that they probably arise during the course of the disease as a result of an epitope spreading phenomenon [69, 70]. Epitope-spreading is defined as the emergence of secondary epitopes on the same or different initial antigen, due to the damage induced in the target tissue by the action of a primary autoantibody [77]. This phenomenon was recently shown to play an important role in the severity and progression of autoimmune diseases including BP, with its both conventional and DPP-4i-induced varieties [78, 79].

From another hand, DPP-4 is known to have a significant role in the immune system through both its enzymatic and non-enzymatic functions. It is expressed in various immune cells such as T cells CD4(+) CD8(+), B cells, macrophages, dendritic cells and NK cells, and is able to modulate their functions. Its role as a potent costimulatory molecule in T cell signal transduction is now well know [36]. Through its catalytic action, DPP-4 can regulate the activity of several cytokines, peptide hormones and chemokines, affecting molecules or signaling pathways pertaining to the immune system. Consequently, the inactivation of DPP-4 by DPP-4i may potentially lead, among other effects, to the breakage of the immune tolerance for the basement membrane antigens, including BP180 and BP230. Inhibition of DPP-4 is thought also to enhance the activity of proinflammatory chemokines like CCL11/eotaxin promoting eosinophil activation in the skin and blister formation [14, 43]. It was indeed shown that blood, skin and blister-derived eosinophils were strongly activated in patients with BP, with increased surface expression of CD69 compared to controls [80]. Moreover, gliptin intake, blocks the transformation of plasminogen into plasmin, and inhibit of the cleavage of BP-180 by plasmin, thus impacting its antigenic properties and/or its function [70].

Ujiie et al. [81] conducted a robust study aimed at finding potential genetic factors that contribute to the pathogenesis of DPP-4i-induced BP. These experts examined HLA alleles in Japanese patients with BP who were on DPP-4i for T2D for at least 3 months before BP onset, and found that 86% of noninflammatory forms carry the allele HLA-DQB1*03:01. Additionally, they demonstrated that frequency of alleles HLA-DQB1*03:01, DQA1*05:05, DRB1*11:01, and DRB1*12:01 was significantly higher, and frequency of alleles HLA-DQA1*01:03 and DQB1*06:01 was significantly lower, in DPP-4i-induced BP than in the Japanese general population. No difference in the frequency of these alleles was found in conventional BP patients compared to the general population. Furthermore 2 alleles HLA-DQB1*03:01 and -DRB1*12:01, were significantly higher in DPP-4i-induced BP patients compared to patients on DPP-4i for 2 years without side effects. The latter statement supports the fact that HLA-DQB1*03:01 could be a potential predictive marker for noninflammatory DPP-4i-induced BP in the Japanese population. However, HLA-DQB1*03:01 was shown to be related to certain subsets of mucus membrane pemphigoid in other populations [82].

### Clinical features

BP induced by DPP-4i seems to appear at earlier age than spontaneous BP. In a study comparing 2 sub-cohorts of DPP-4i-related BP and spontaneous BP, the median age of onset was 77.5 years (59.0–94.0) for the induced BP and 82.0 (56.0–95.0) for the spontaneous form [14]. Across a multitude of case reports, the mean age of onset for gliptin-induced BP ranges from 72.5 to 80 years, however cases in patients aged 60 years or below have been reported [10, 51].

The latency between the start of the treatment with DPP-4i and the onset of BP symptoms varies widely across studies. According to several recent observations, the latency interval varies from 8 days to 6.5 years [33, 34, 62, 67].

Most of the recent European studies do not report majors distinctives clinical features between spontaneous and DPP-4i-induced BP [16]. Generally, in a similar way than conventional BP, DPP-4i-induced BP manifests as a diffuse bullous eruption of the trunk, extremities and face, comprised of tense bullae progressing into erosions and crusts. Blisters appear mostly over an erythematous and edematous base, and are accompanied by an intense pruritus. However, in several recent case series, patients with DPP-4i-induced BP tend to manifest a noninflammatory phenotype, distinct from conventional BP [83]. In this noninflammatory form, bullae appear mostly over a normal appearing skin, and the eruption is comprised of a few, mild erythematous lesions with limited distribution [68, 69, 74, 82]. An exceptionally localized form of BP manifesting as a recurrent single blister in the upper limb, was reported in association with gliptin exposure [84]. Nonetheless, the classical inflammatory type similar to spontaneous BP is also often seen in the majority of case series, suggesting that DPP-4-i-related BP may manifest either as inflammatory or noninflammatory phenotype [55, 69, 81].

Using the standardized scoring for BP the Bullous Pemphigoid Disease Area Index (BPDAI) [85], Ujiie et al. [81] showed that BP induced by DPP-4i is heterogenous, and may belong to 2 distinct clinical subcategories, an inflammatory forms, with a BPDAI for erythema/urticaria ≥ 10 and noninflammatory forms with a BPDAI for erythema/urticaria < 10. Furthermore, these authors showed that BPDAI erythema/urticaria scores were significantly higher in conventional BP with diabetes than in DPP-4i-induced BP, suggesting that the noninflammatory phenotype is associated to the intake of DPP-4i and not to the existence of T2DM. BPDAI score for erosion/blisters was found similar in conventional and gliptin-induced BP. Conversely, Chijiwa et al. showed that BPDAI scores were similar between gliptins-induced and spontaneous BP, except for erosions/blisters score in mucosa which was significantly higher in gliptins-induced BP [86]. Nonetheless, mucosal involvement, seen in 10–30% patients with conventional BP [3, 55], is not well investigated in gliptins-induced BP, but it seems that diabetic patients with DPP-4i-induced BP tend to manifest more mucosal involvement than their diabetic counterparts on other treatment regimen [14]. Finally, to the best of our knowledge, atypical forms of BP, manifesting as urticaria-like, prurigo-like, eczema-like, infiltrated plaques, and which account for 20% of conventional BP [87, 88], were never reported in association with DPP-4i. Through a comprehensive literature review on DIBP, only a series of 3 patients with erythema multiforme-mimicking form of BP after penicillin exposure were reported [5] but no cases of atypical BP related to DPP-4i were observed.

### Histopathological and immunological profiles

Gliptin-induced BP shares the same histopathological and immunofluorescence profiles than spontaneous BP [16, 55]. The hallmark histological sign is the presence of subepidermal blisters or a sub-epidermal detachment. The blister cavity may contain numerous eosinophils, neutrophils and fibrin. A dense polymorphous dermal inflammatory infiltrate containing neutrophils, lymphocytes, histiocytes with eosinophil predominance is usually present [3, 4]. However, Chijiwa et al. reported recently a distinctive histological characteristic in DPP-4i-related BP, which is a significantly lower number of eosinophils infiltrating the upper dermis of peri-blisterlesions, in patients treated by DPP-4i compared to patients with spontaneous BP. This histological feature was seen in specific clinical forms which belong to the noninflammatory phenotype [74, 86]. The direct immunofluorescence (DIF) study, done in normal-appearing perilesional skin, demonstrates IgG and complement C3 deposition in a linear band at the dermal–epidermal junction: these finding are identical in both DPP-4i induced and conventional BP and area paramount diagnostic feature. Indirect immunofluorescence (IIF) documents the presence of IgG circulating autoantibodies in the patient's serum by showing on human salt-split skin IgG staining on the epidermal side of the blister [3, 54]. The IIF aspect in DPP-4i-induced BP is indistinguishable from that seen in spontaneous BP.

Quantification of antibodies anti-BP180 and anti-BP230 using ELISA, have not shown, so far, a significant difference in the titer of autoantibodies, between spontaneous and DPP-4i-induced BP [85]. However, Kinyó et al. reported recently that the antibodies titer is lower in DPP-4i-induced B.

The specificity of these autoantibodies, may be different in both forms. While 80–90% of patients with spontaneous BP are positive for anti-BP180 NC16A autoantibodies, a significant proportion of patients with DPP-4i-related BP, estimated at 40–70% in a Japanese study [16], have negative BP180-NC16A ELISA, and their autoantibodies rather recognize other epitopes, midportion of the BP180 ectodomain and/or C-terminal domain of BP180 [55, 74]. In patients with DPP-4i-induced BP, the absence of BP180-NC16A antibodies corresponds to a non-inflammatory clinical form of BP [16, 74, 86] while the positivity for BP180-NC16A is more present in inflammatory forms that in noninflammatory ones [81].

Finally, blood eosinophilia, present in around 50% of conventional BP patients [87], and reported since long as a marker of the disease severity [89, 90], seems to be less common and less significant in patients with DPP-4i-related BP [15, 83]. Besides that, other blood-based markers for BP, like the increased amount of soluble IL-2 receptor, macrophage migration inhibitory factor, eosinophilic cationic protein, neutrophil-derived myeloperoxidase (elevated in sera and blister fluids) and mast cell degranulation assays [91] were not studied in DPP-4i-related BP in comparison with spontaneous BP, and their use in clinical settings is very limited.

### Treatment and clinical outcome

As for spontaneous BP, treatment of DPP-4i aims to prevent the development of new skin lesions, to allow cutaneous healing and to control the pruritus. There are no specific guidelines for the treatment of such condition and most data on treatment of gliptins-associated BP come from isolated case reports. The need for a prompt withdrawal of the offending drug is unanimously admitted. In the vast majority of patients, definitive withdrawal of the culprit drug followed by high-potency topical steroids only or in association to low doses of systemic steroids achieve clinical remission [3, 10, 33, 34, 61, 67]. A Spanish study reported a positive outcome for all observed patients: the withdrawal of the culprit DPP-4i was sufficient to obtain resolution of all symptoms in a small number of patients, and a minimal steroid systemic treatment at low doses and short duration was needed for the remaining cases [55]. In another small cohort, patients showed persistent cutaneous symptoms despite the administration of steroids and improvement was obtained only after cessation of DPP‐4i [14]. In a case of BP induced by vildagliptin, the introduction of topical clobetasol, induced a resolution of the symptoms despite the continuation of the causative drug, but the skin lesions relapsed 3 months later and complete remission was achieved only after definitive cessation of vildagliptin [59]. Generally, high-potency topical steroid (0.05% clobetasol cream, 10–30 g/day topical) and/or systemic steroid (prednisone) at low dose (0.5 mg/kg/day) are needed to obtain disease control. The dose of oral corticosteroids is tapered after suppression of inflammation and blistering, and generally no risk of relapse is expected once the offending drug is stopped. Exceptionally, switch to intravenous immunoglobulin is needed to promote disease control, mainly in debilitated patients [5].

### Bullous pemphigoid and the need for further research

In light of the reviewed data, it seems that BP associated with DPP‐4i may present as 2 distinct forms with different clinical, histological and immunological profiles. The first form features widespread inflammatory skin lesions and blisters, has positive autoantibodies to BP180‐NC16A and is indistinguishable from the spontaneous BP. The second form is non‐inflammatory, it is characterized by milder, less diffuse and less inflammatory skin lesions, negative autoantibodies against BP180‐NC16A, and less eosinophil infiltrate on skin biopsy. These second form seems to be associated to a specific HLA types, like the HLA-DQB1*03:01 in the Japanese population. This latter form may progress into the inflammatory form by epitope spreading mechanism. The relative prevalence of both forms varies from one observation to another, the noninflammatory form is predominant in certain studies [79]. To our view, distinction should be done between the 3 forms of BP: the spontaneous BP, the inflammatory DPP-4i-related BP, and the noninflammatory DPP-4i-related BP. Understanding their shared and distinct pathogenic mechanisms would be relevant for the prevention of the latter forms. Further research, based on integrated epidemiological, clinical, histo-immunological and pharmacogenomic approaches, may give more insight into these skin conditions. This combined approach will allow to better define BP endotypes, to unveil the mechanism of spontaneous or drug induced breakage of the immunotolerance to skin self-antigens, including the epitope spreading phenomena and its role in sustaining the autoimmunity in BP.

## Conclusion

Given the increasing numbers of T2DM patients worldwide, and the many advantages offered by gliptins in the management of this disease, it is expected that the prescription of gliptins will rise accordingly. Despite the increasing evidence about the responsibility of DPP-4i in the occurrence of BP, to this point, no clear individual risk factors have been identified that help predict the susceptibility of developing BP in patients treated by DPP-4i. Additionally, the chronology between the exposure to DPP-4i and the onset of BP is variable, therefore, any diabetic patient on gliptins should be considered at risk of developing BP and certain gliptins such as vildagliptin should be used cautiously. Further research to understand the molecular mechanistic action of gliptin in BP, and to identify high risk patients is warranted.

## Data Availability

Not applicable.

## Abbreviations

BP: Bullous pemphigoid
DPP-4i: Dipeptidyl peptidase-4 inhibitors
DIF: Direct immunofluorescence
BP-180: Bullous pemphigoid antigen BP180
BP-230: Bullous pemphigoid antigen BP230
ELISA: Enzyme-linked immunosorbent assay
T2DM: Type 2 diabetes mellitus
DIBP: Drug induced bullous pemphigoid
ACE: Angiotensin-converting enzyme
CD26: T-cell activation antigen CD26
GLP-1: Glucagon-like peptide-1
GIP: Glucose-dependent insulinotropic polypeptide
DPP-8: Dipeptidyl peptidase 8
DPP-9: Dipeptidyl peptidase 9
BPDAI: Pemphigoid Disease Area Index
IIF: Indirect immunofluorescence
IL-2: Interleukin-2
